# Precise Point Positioning with the BeiDou Navigation Satellite System

**DOI:** 10.3390/s140100927

**Published:** 2014-01-08

**Authors:** Min Li, Lizhong Qu, Qile Zhao, Jing Guo, Xing Su, Xiaotao Li

**Affiliations:** GNSS Research Center, Wuhan University, No.129 Luoyu Road, Wuhan 430079, China; E-Mails: limin@whu.edu.cn (M.L.); zhaoql@whu.edu.cn (Q.Z.); whuguojing2005@163.com (J.G.); longtian00@163.com (X.S.); lixiaotao@whu.edu.cn (X.L.)

**Keywords:** BeiDou navigation satellite system, Position and Navigation Data Analyst (PANDA), BeiDou Experimental Tracking Stations (BETS), Precise Point Positioning (PPP)

## Abstract

By the end of 2012, China had launched 16 BeiDou-2 navigation satellites that include six GEOs, five IGSOs and five MEOs. This has provided initial navigation and precise pointing services ability in the Asia-Pacific regions. In order to assess the navigation and positioning performance of the BeiDou-2 system, Wuhan University has built up a network of BeiDou Experimental Tracking Stations (BETS) around the World. The Position and Navigation Data Analyst (PANDA) software was modified to determine the orbits of BeiDou satellites and provide precise orbit and satellite clock bias products from the BeiDou satellite system for user applications. This article uses the BeiDou/GPS observations of the BeiDou Experimental Tracking Stations to realize the BeiDou and BeiDou/GPS static and kinematic precise point positioning (PPP). The result indicates that the precision of BeiDou static and kinematic PPP reaches centimeter level. The precision of BeiDou/GPS kinematic PPP solutions is improved significantly compared to that of BeiDou-only or GPS-only kinematic PPP solutions. The PPP convergence time also decreases with the use of combined BeiDou/GPS systems.

## 1. Introduction

As a global navigation satellite system (GNSS) compatible with other worldwide navigation satellite systems, the BeiDou System (BDS) was independently established and operated by China. High accuracy and high reliability positioning, navigation and timing (PNT) services are expected to be provided to all kinds of users in any time, all-weather and anywhere in the World by 2020. Until the end of 2012, the constellation of BeiDou regional navigation satellite system consists of 16 satellites including six GEOs, five IGSOs and five MEOs with 14 satellites including five GEOs, five IGSOs and four MEOs operating well. The test version of Interface Control Document (ICD) for the signal-in-space of the BeiDou system was announced on December 27, 2011. This indicated that the BeiDou system started to provide Initial Operational Service. The formal version of ICD was announced and the Full Operational Service was provided for China and its surrounding areas on December 27, 2012. This indicated that the regional navigation satellite system had been set up. Domestic and foreign enterprises are being encouraged to participate in the research and development of BeiDou system application terminals (see http://www.BeiDou.gov.cn).

Since M01, the first satellite of BeiDou-2 system, was successfully launched, there have been various research projects studying the signal structure, the precise orbit determination of BeiDou satellites and the precise positioning performances. The signals of M01 have been tracked and the structure of the pseudo-random noise (PRN) code in the E2 (B1), E5b (B2), and E6 (B3) frequency bands has been analyzed [1,2]. The signal power of BeiDou M01 satellite has been found larger than that of GPS satellites and the GIOVE-B satellite of Galileo. Based on the “BETS” and the PANDA software, the precise orbit determination of the BeiDou satellites and PPP by using the precise orbit and satellite clock bias products have been investigated. The RMS values of the overlap differences in the radial component reached 10 cm and the daily static PPP BeiDou solutions showed an accuracy of 2 cm in horizontal components and 7 cm in the vertical component [3]. Satellite Laser Ranging (SLR) tracking data have been used to determine the orbit of BeiDou M01 with 7 days arc length. The RMS values of the 3-D overlap error varied from 11 to 50 cm in the selected periods [4]. The performances of the static and kinematic precise relative positioning have been also analyzed. The precision of static precise relative positioning solutions reached millimeter level and the kinematic solutions reached centimeter level [5]. It should be mentioned that the positioning results above were acquired when there were just six BeiDou satellites. With the increasing number of satellites, more research is being conducted on the BeiDou system. As a result of the fewer tracking stations and the lower precision of BeiDou orbit and clock biases product, the biases in the horizontal and vertical components of static PPP reached 12 cm [6]. The German Research Center for Geosciences (GFZ's EPOS-RT software package obtained similar results to those of the PANDA software [7]. A BeiDou PPP based on the products from a small network of six stations also provided an accuracy of several centimeters compared to the GPS-only results [8]. To improve the satellite orbits of the BeiDou regional system, the impact of the tracking geometry, the involvement of MEOs and the effect of integer ambiguity resolution were analyzed as well [9]. Meanwhile, the capacity of the BeiDou system for tropospheric remote sensing was also assessed. The experiment showed that the derived Zenith Tropospheric Delay (ZTD) was close to GPS ZTD estimated using different software packages [10].

The improvement of performance of a single Global Navigation Satellite System (GNSS) mainly depends on increasing the number of navigation satellites, optimizing the spatial geometric configuration and improving of the accuracy of the system's spatial signals, including the improvement of the accuracy of the precise orbit and satellite clock biases, whereas, the fusion of multiple GNSSs can significantly increase the number of observed satellites, optimize the spatial geometry and improve continuity and reliability of positioning [11]. However, in the past the data processing of multi-GNSS focused on the fusion of GPS and GLONASS [11–14]. By processing the simulated data, the contribution of the BeiDou navigation satellite system to global PNT users has been analyzed in [15], which demonstrated that the introduction of the BeiDou system could benefit significantly the satellite visibility and the dilution of precision for global users. Research on the BeiDou positioning and combined BeiDou/GPS positioning will obviously benefit the application and popularization of the BeiDou system. With the orbit and satellite clock biases derived based on a small network of six stations distributed in Asia-Pacific area, a centimeter level PPP was realized by combined BeiDou and GPS data [16]. The impact of “inter-satellite-type-bias” (ISTB) was also analyzed when multiple GNSS receivers were used to determine the carrier's attitude [17]. In this contribution, we also adapted the PANDA software developed by Wuhan University to process the BeiDou/GPS observations of “BETS” in the modes of BeiDou static and kinematic PPP, GPS static and kinematic PPP and BeiDou/GPS static and kinematic PPP.

## 2. Quality Analysis of BeiDou Measurements

**Figure 1.**
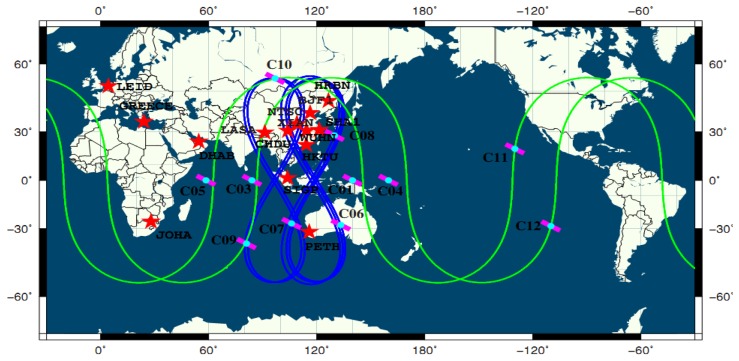
The distribution of “BETS” and the tracks of the sub-satellite points of four GEO, five IGSO and two MEO BeiDou Satellites.

In order to obtain the precise orbits and satellite clock biases of BeiDou satellites to assess the performance of precise positioning, since 2011 Wuhan University has established a continuous worldwide observation reference network, called the BeiDou Experimental Tracking Stations (BETS), which includes nine tracking stations in China and six tracking stations abroad. All the stations are equipped with UB240-CORS receivers produced by the Beijing Unicore Communication Company, which can acquire the pseudo-range and phase observations of dual frequencies and dual systems. The Unicore UA240 dual-frequency dual-system high gain antenna is used in the network. The accuracy of phase observations can reach millimeter level [6]. The GEO C02 and MEO C30 did not work and were discarded [18]. The MEO C13 and C14 are not included as they were still under in-orbit testing. The GEO C15 was also under in-orbit testing and is planned to replace C02. Hence, 11 BeiDou satellites were employed for data processing in this contribution. They were four GEOs including C01, C03, C04 and C05, five IGSOs including C06–C10 and two MEOs including C11 and C12. Figure 1 shows the distribution of BETS around the world and the tracks of satellite points of the BeiDou system. As Figure 1 shows, the ground tracks of the five BeiDou IGSOs are confined from approximately 55°S to 55°N latitude and 102°E to 135°E longitude. They describe two “8” shape loops with a mean longitude difference of roughly 30°, so the eastern and western parts of China and the adjoining regions can be covered effectively. There are four GEOs distributed in the Indian and Pacific oceans over the Equator as supplements for the IGSO satellites to ensure users in Asian-Pacific regions can observe enough satellites. They are located at the 58.75°E, 80.0°E, 140.0°E, and 160°E longitudes, respectively. The inclination of the orbit of a BeiDou MEO is about 55°, and the orbit height is between that of GPS and Galileo satellites [19]. The revolution period is about 773 minutes, leading to 1.86 revolutions per day. The ground track of the satellite repeats every seven days [4].

### 2.1. Satellite Visibility

**Figure 2.**
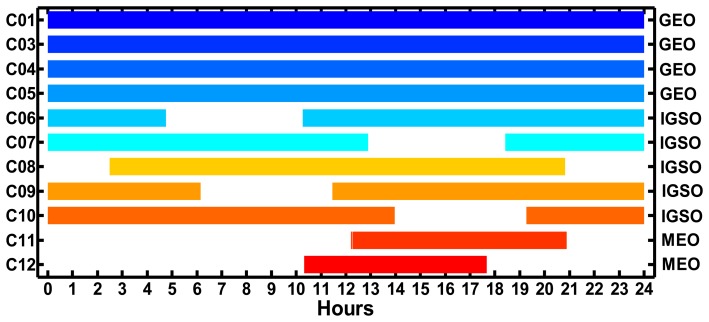
Visibility of BeiDou satellites at CHDU on September 24, 2012 (the mask elevation is 7°).

Figure 2 shows the visibility of BeiDou satellites at CHDU on September 24, 2012. It can be seen from the figure that four GEO satellites C01, C03, C04 and C05 are tracked all day long, while five IGSO satellites C06–C10 are not tracked continually without breaks for 24 h. The lengths of the data gaps for all the IGSO satellites are about 4.5 h. Two MEO satellites (C11 and C12) are tracked in a shorter time than that of GEO and IGSO satellites. It is also seen that at least three IGSO satellites are tracked at every moment so at least seven BeiDou satellites are tracked for every epoch, which guarantees the realization of BeiDou PPP.

**Figure 3.**
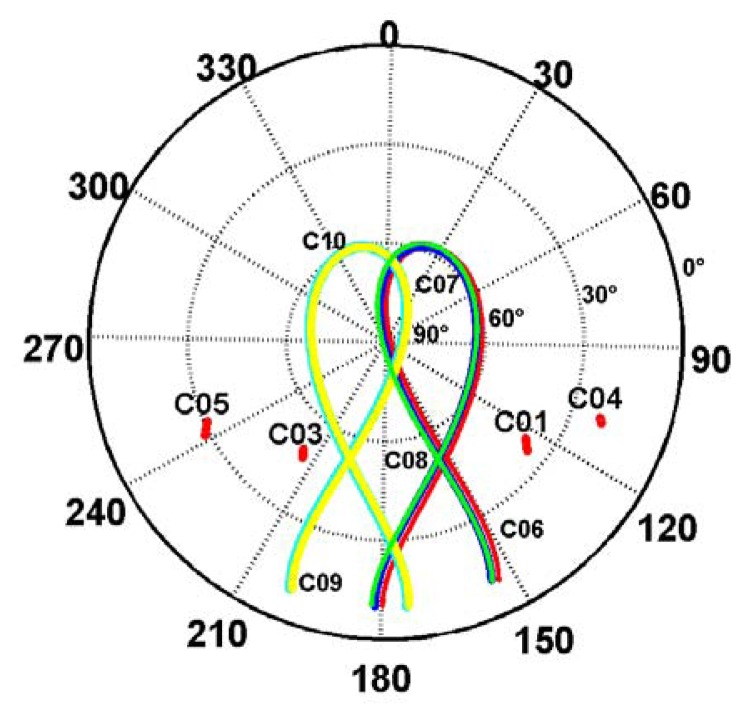
Sky plots (azimuth *vs.* elevation) of five IGSO and four GEO satellites at CHDU on September 24, 2012.

Figure 3 shows the sky plots of five IGSO and four GEO satellites against the azimuths and elevations. It can be seen that the elevations and azimuths of C01 are within 30–45° and within 120°–135° and that of C03 are within 45°–60° and within 210°–225°, respectively. The elevations of C04 and C05 are lower than that of C01 and C03, as they are further away from the station. Meanwhile, the five IGSO satellites have a ground track of an incomplete “8” shape as their elevations are below the mask angle and the satellites cannot be observed. Furthermore, we can find that for mainland China, the BeiDou GEO and IGSO satellites cover the southern hemisphere most of the time.

### 2.2. Analysis of Multipath and Noise

The multipath combination (MPC) is often used to assess the multipath and code noise level of a receiver [4–6]. The MPCs eliminate the first-order ionosphere delay and the geometry distance between satellites and stations. The systematic error and the noise of carrier-phase usually could be neglected compared with that of the code observations. Then the MPCs contain a constant ambiguity term of combined carrier phases, systematic hardware delay and thermal noise plus noise of code measurements. The MPCs for the receiver and satellites on two frequencies can be expressed as [4–6]:

(1) $$\text{MPC}1_{r}^{s}=P_{1r}^{s}-(a+1)\cdot B_{1r}^{s}+a\cdot B_{2r}^{s}$$

(2) $$\text{MPC}2_{r}^{s}=P_{2r}^{s}-b\cdot B_{1r}^{s}+(b-1)\cdot B_{2r}^{s}$$

where $a=2f_{2}^{2}/(f_{1}^{2}-f_{2}^{2})$, $b=2f_{1}^{2}/(f_{1}^{2}-f_{2}^{2})$, *P* and *B* are the BeiDou code and phase observations, respectively; *f*_1_ and *f*_2_ are the frequencies of *B*_1_ and *B*_2_.

**Figure 4.**
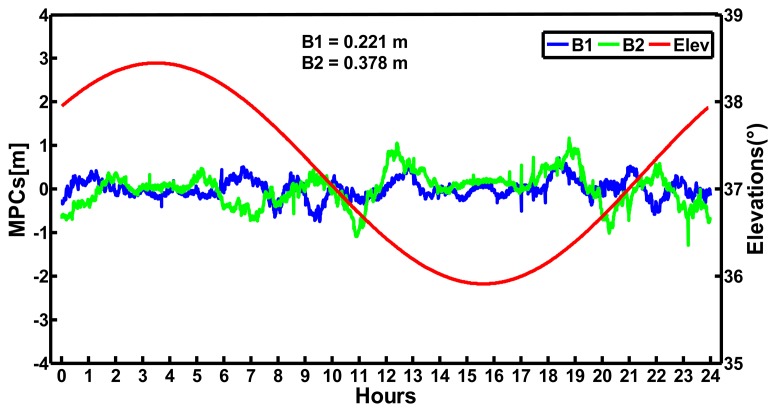
MPCs of the B1 and B2 codes, as well as the variation of the elevations of satellite C01 (GEO) at CHDU station on September 24, 2012.

We processed the data of the CHDU station on September 24, 2012 to analyze the multipath and noise error. The station is equipped with the Unicore UB240-CORS receiver and UA240 antenna. The mask elevation is 7° and the interval is 30 s. Figure 4 displays the MPCs of the B1 and B2 codes and the variation of the elevations of satellite C01 at CHDU on September 24, 2012. It can be seen that the variations of the elevations of C01 are within 35°–39°. The MPCs of B1 and B2 codes of satellite C01 have small changes with the variations of the elevations. The MPCs of B1 codes are steadier than that of B2 codes and the standard deviation of the MPCs of B1 codes is smaller than that of B2 codes. Furthermore, both the MPCs of the B1 codes and B2 codes are within ±1 m.

**Figure 5.**
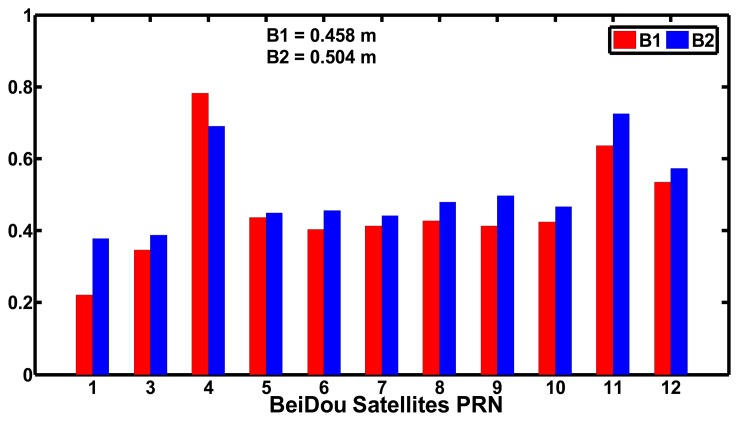
Standard deviations of MPCs of B1 and B2 codes of BeiDou satellites at CHDU station on September 24, 2012.

Figure 5 shows the standard deviations of the MPCs of the B1 and B2 codes of the BeiDou satellites. The standard deviations of MPCs of B1 and B2 codes of the BeiDou satellites are smaller than 0.5 m, except for C04, C11 and C12 and the average values are 0.458 and 0.504 m, respectively. The standard deviations of MPCs of B1 codes are smaller than that of B2 codes, except for C04. We also can see that the MPCs are different for different types of satellites. The GEO satellites, except for C04, have the best performance while the MEO satellites have the worst performance.

**Figure 6.**
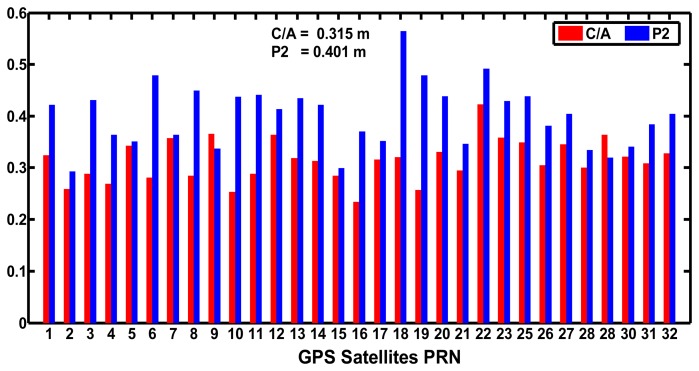
Standard deviations of MPCs of C/A and P2 codes of GPS satellites at CHDU station on September 24, 2012.

Figure 6 shows the standard deviations of the MPCs of C/A and P2 codes of GPS satellites. The standard deviations of C/A and P2 codes of all the GPS satellites are less than 0.50 m except for the G18 satellite. The average values of the standard deviations of the C/A codes of all the GPS satellites are 0.315 m, which is smaller than that of the B1 codes of BeiDou satellites. The average standard deviations of P2 codes are 0.401 m, smaller than that of the BeiDou B2 codes. The results above show that the accuracy of GPS code observations at the CHDU station is better than that of the BeiDou code ones. The values do not mean that BeiDou code observations are worse than GPS ones. The reason may be that the MPCs derived based on Equations (1) and (2) were composite values that not only include the pseudo-range multipath error but also other errors, such as the pseudo-range and phase noise, and the hardware delay in the receivers and satellites. In addition the BeiDou system constellation is not complete, but the GPS statistics contain all satellites of the constellation.

## 3. BeiDou PPP Strategy

The Position and Navigation Data Analyst (PANDA) software package, developed by the GNSS Research Center at Wuhan University, was used to process the BeiDou/GPS observations of “BETS”. Since 2003, the functions of the software have been extended continuously. PANDA software can be applied in the precise orbit determination for the GPS, GLONASS, the low-orbit satellites, PPP, PPP-RTK, the network adjustment, the precise relative positioning as well as the gravity field modeling using satellite-to-satellite tracking data [20–26]. Nowadays, the PANDA software package has been further developed to process the BeiDou data with the capablility of precise orbit and clock determination, as well as precise positioning [3,5,27].

The PPP parameters to be estimated contain the coordinates, the receiver clock biases, the delay of the wet troposphere component and the ambiguities when only processing BeiDou data. When BeiDou/GPS observations are processed in a combined mode, we need to introduce an inter-system bias between multiple GNSS signals in the receiver end [11–14].

BeiDou precise orbit and satellite clock bias products used in the contribution are based on the ITRF2008 reference frame, which is the same as used in single GPS data processing [3]. The orbit and satellite clock biases were generated using the strategy proposed by Zhao *et al.* [27]. In data processing, we used the Turbo-Edit method to detect the major cycle slips and eliminate the major outliers in the first stage. In the second stage, the residuals-editing method is used to detect the minor cycle slips, and eliminate remaining outliers [28]. The cutoff elevation of 7° and the variance component estimation weighting method were applied. We consider corrections related to the satellites (*i.e.*, the default satellite antenna phase offset from the BeiDou satellites and the satellite antenna phase offset and PCV of GPS satellites), the corrections related to the propagation path (*i.e.*, the zenith troposphere delay, the phase wind up), and the corrections related to the ground stations (*i.e.*, the solid earth tide, the ocean loading tide. However, the phase center variation (PCV) of BeiDou satellites and the PCO and PCV corrections in the receiver side are not considered. The reason is that those values are not acquirable at present. The parameters estimation strategies and detail corrections are listed in Table 1.

## 4. Results and Discussion

In order to assess the precision of BeiDou PPP solutions, we processed 27 days' data from September 4 to September 30 in 2012 (day of year from 248 to 274) by using the PANDA software package in the modes of BeiDou, GPS, and BeiDou/GPS static daily PPP solutions. Among the stations used below in Table 2, CHDU was not used for orbit and clock determination, so the solutions are independent.

### 4.1. Static PPP

Table 2 shows the biases of the results of the BeiDou, GPS, and BeiDou/GPS static PPP against the “ground truth” in the East, North and Up components on September 24, 2012. The “ground truth” is the mean value of the GPS static PPP coordination from September 4 to September 30, 2012. We can see that the biases in the East, North and Up components vary from station to station. Stations in the Asia-Pacific regions have better accuracy than stations in Europe like Greece and LEID. The biases of the East and North components of the stations in the Asia-Pacific region are almost within ±1 cm except that of the East component of the LASA station, whose absolute value of the vertical bias reaches 4 cm. Meanwhile, the biases of stations in Europe, especially the GREECE station, are even worse. However, the biases of the three components of GPS PPP solutions for all the stations are almost within ±1 cm, better than that of BeiDou. That is because the “ground truth” represents the average values of GPS static PPP. The horizontal accuracy of BeiDou static PPP are closer to that of GPS than the Up components. In addition, the PCV corrections of BeiDou satellites are not applied, and the PCO corrections of BeiDou satellites are not accurate enough. Furthermore, the biases between GPS and BeiDou signal phase center for the unknown PCO and PCV corrections of the receivers could introduce biases into the results.

**Figure 7.**
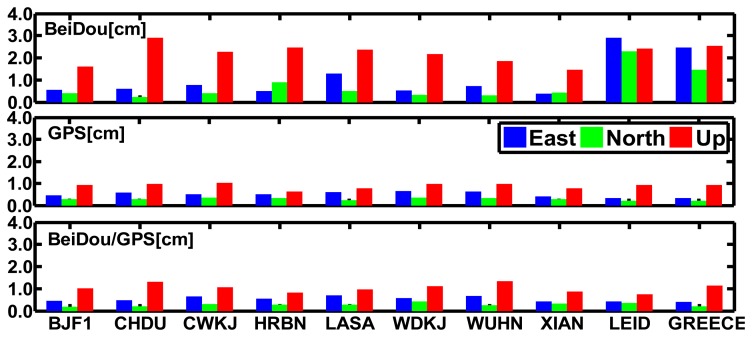
RMS values of the differences between the BeiDou, GPS, and BeiDou/GPS static PPP results and the “ground truth” in the East, North and Up components from September 4 to September 30 in 2012.

Figure 7 shows the RMS values of the daily biases for the BeiDou, GPS and BeiDou/GPS static PPP solutions of the “BETS” network with respect to the “ground truth” in the East, North and Up components from September 4 to September 30, 2012. We can see that:

- The RMS values of the East, North and Up components also show spatial variations. The RMS values of three components of BeiDou static PPP against the “ground truth” are less than 1.0 cm, 1.0 cm and 3.0 cm in the Asian-Pacific in the period. However, the results get worse for the LEID and GREECE stations, which are far from the Asia-Pacific region. Their RMS values are less than 4.0 cm due to the fewer BeiDou satellites observed as shown in Figure 1. The average values of the RMS of the East, North and Up components for all the stations are 1.07, 0.73 and 2.21 cm, respectively.
- The RMS values of the difference between the East, North and Up components of BeiDou/GPS static PPP and the “ground truth” for all the stations are almost all less than 1.0 cm and the average values of the RMS values are 0.53, 0.28 and 1.04 cm, respectively. Those of GPS static PPP for all the stations are 0.50, 0.29 and 0.89 cm, respectively. The results illustrate that the accuracy of GPS static PPP do not improve obviously with the introduction of BeiDou data. Probably, the orbit error of BeiDou satellites may still have biases with respect to that of GPS satellites.

### 4.2. Kinematic PPP

**Figure 8.**
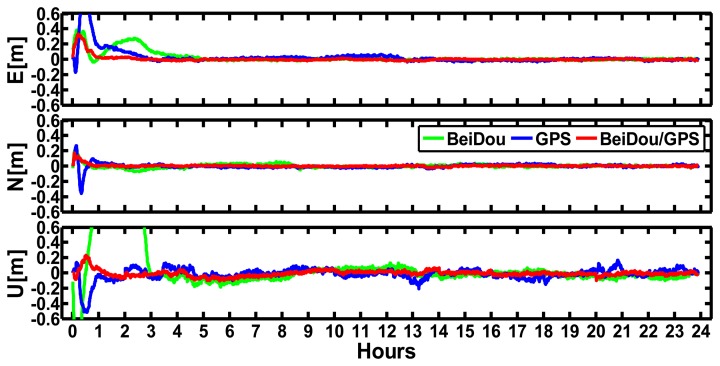
Differences between BeiDou, GPS, and BeiDou/GPS kinematic PPP solutions with respect to the “ground truth” in the East, North and Up components of CHDU station, respectively on September 24, 2012.

**Figure 9.**
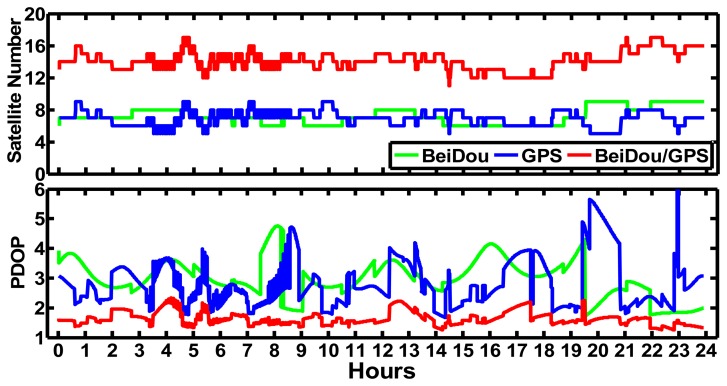
Variations of satellite numbers used and PDOP of the BeiDou, GPS, and BeiDou/GPS of CHDU on September 24, 2012.

The kinematic PPP performance was also assessed for the modes of BeiDou-only, GPS-only and BeiDou/GPS by processing the daily data from September 24 to September 30 in 2012 at CHDU station. The interval is 30 s. We analyzed the precisions of BeiDou-only, GPS-only and BeiDou/GPS kinematic PPP solutions after convergence. The solutions are compared with the “ground truth” of CHDU station. Meanwhile, the convergence time was also analyzed. Figure 8 shows the difference of the results of the BeiDou, GPS and BeiDou/GPS kinematic PPP at CHDU station against the “ground truth” in the East, North and Up components on September 24, 2012. Figure 9 shows the satellite numbers and PDOP variations on that day. We can find the following results:

1. The north component of BeiDou-only kinematic PPP converges faster than the East and Up components at CHDU station. The coordinates difference against the “ground truth” for the North and East components of BeiDou-only kinematic PPP after convergences are within ±10 cm while that of the up component are within ±20 cm.
2. The north component of BeiDou-only kinematic solutions converge as fast as that of GPS-only while the East and Up components converge more slowly than that of GPS-only, but as Figure 8 shows, the BeiDou/GPS kinematic PPP converges faster than both BeiDou-only and GPS-only kinematic PPP, especially in the East and North components.
3. As Figure 9 has shown, at least five satellites can be seen at CHDU in every epoch for BeiDou kinematic PPP and the variation PDOP is from 2 to 6. The satellite numbers of combined BeiDou and GPS positioning increase significantly and at least 12 satellites are used in every epoch. The PDOP decreases obviously than that of BeiDou-only and GPS-only cases. As a result, BeiDou/GPS kinematic PPP shows better precision and shorter convergence time than that of BeiDou-only and GPS-only kinematic PPP.

**Figure 10.**
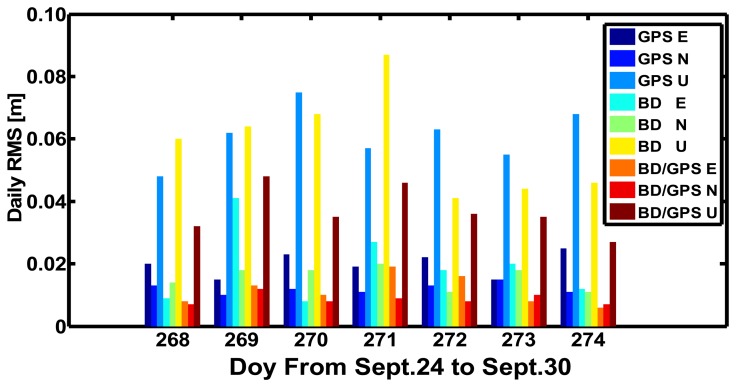
Daily RMS values of BeiDou, GPS, and BeiDou/GPS kinematic PPP solutions of CHDU against the “ground truth” in the East, North and Up components from September 24 to September 30 in 2012 (Day of year is from 268 to 274).

Figure 10 displays the daily RMS values of the results of the BeiDou, GPS, and BeiDou/GPS kinematic PPP in CHDU against the “ground truth” in the East, North, and Up components from September 24 to September 30 in 2012. We can find the following results:

(1) The accuracy of the horizontal components of BeiDou-only kinematic PPP is within 1.0–2.0 cm except DOY 269 and the accuracy of the vertical component is within 4.0–7.0 cm except DOY 271. The average values of BeiDou-only kinematic PPP in the East, North and Up components are 1.93, 1.57 and 5.86 cm respectively, and those of GPS-only kinematic PPP are 1.99, 1.21 and 6.11 cm, respectively.
(2) The accuracy of the horizontal components of BeiDou/GPS kinematic PPP is within 1.0–2.0 cm and that of the vertical component is within 2.0–5.0 cm. The average values of BeiDou/GPS kinematic PPP solutions are 1.14, 0.87 and 3.70 cm, respectively, which significantly improve compared to that of BeiDou-only and GPS-only kinematic PPP, thanks to the increasing of satellite numbers and the improvement of the PDOP.

### 4.3. Residual Analysis

**Figure 11.**
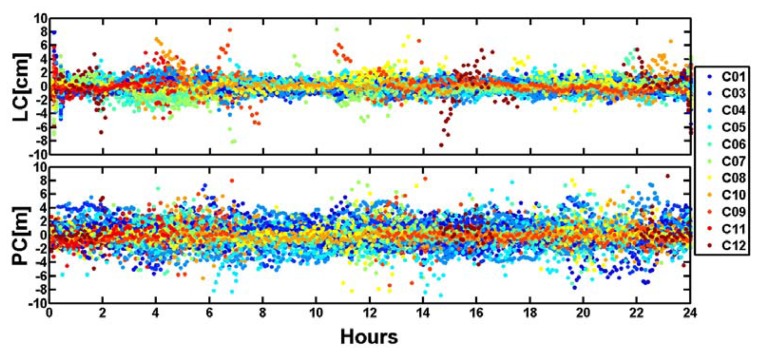
Undifferenced LC and PC residuals of BeiDou satellites.

Observations residuals mainly contain the observation noises, multipath errors, orbit errors and mismodelled errors so that the residuals can be used as an important index to assess positioning accuracy [23–27]. Figure 11 shows the residuals of BeiDou satellite ionosphere-free undifferenced phase (LC) and code (PC) observations residuals of 10 stations of “BETS” on September 24, 2012 (DOY is 268). It can be seen that the LC residuals are nearly within ±2.0 cm and the PC residuals are within ±4.0 m, but because the weighting of code observations is very small compared with that of phase observations, it does not influence the final results significantly.

**Figure 12.**
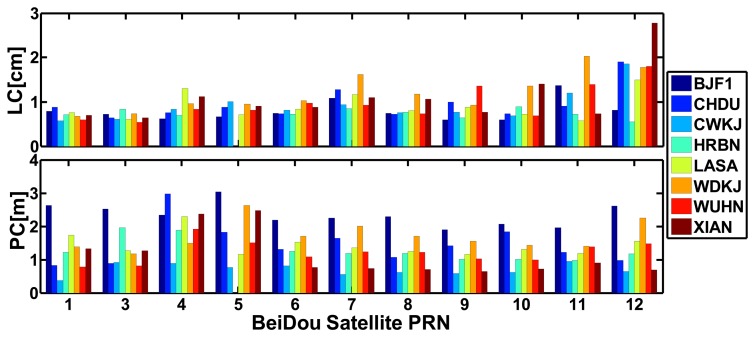
RMS values of undifferenced LC and PC residuals for the BeiDou satellites of all individual sites.

**Figure 13.**
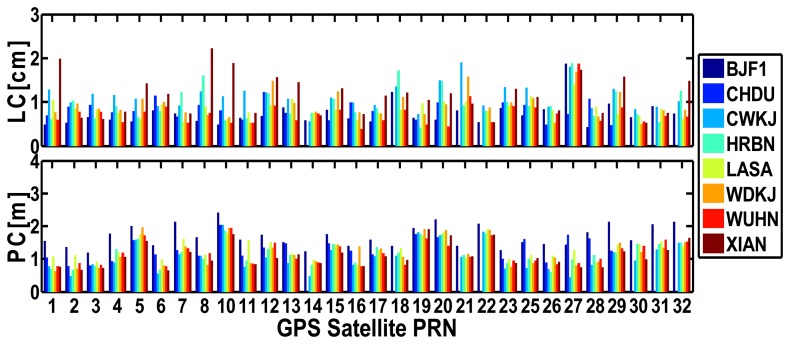
RMS values of the undifferenced LC and PC residuals for GPS satellites of all individual sites.

Figure 12 shows the RMS values of the residuals of BeiDou satellites for each site of “BETS” on September 24, 2012. The average RMS values of undifferenced LC and PC residuals are listed in Table 3. Figure 13 shows the RMS values of the residuals of GPS satellites for “BETS” on September 24, 2012. On the basis of these figures and table, we can find that:

(1) The RMS values of the PC residuals of BeiDou satellites for “BETS” are smaller than 3 m and the RMS values of the LC residuals of GEO and IGSO satellites are smaller than 1.5 cm while those of MEO satellites are within 1–3 cm. By contrast, the RMS values of the LC and PC residuals of GPS satellites have the similar accuracy. The RMS values of the LC and PC residuals of GPS satellites are smaller than 2 cm and 2 m, respectively. The average values of the RMS of the undifferenced LC and PC residuals of BeiDou satellites for “BETS” are 0.95 cm and 1.42 m, respectively. And the average values of the RMS of the undifferenced LC and PC residuals of GPS satellites are 0.91 cm and 1.25 m, respectively. That BeiDou system has larger LC and PC residuals than GPS may be caused by the multipath error.
(2) The RMS values of the residuals of GEO C01 and C03 are smaller than that of GEO C04 and C05 and the IGSOs, while those of the MEOs are the worst. This may be due to the fact that the elevations of GEO C01 and C03 are larger than those of other satellites and also do not change much, which leads to a smaller multipath error. Meanwhile, the orbit accuracy of GEO C01 and C03 is better than that of GEO C04 and C05 [3]. Furthermore, because the PCV of BeiDou satellites are not considered and the PCO may be not accurate enough, there may be a satellites-specific bias in the residuals. In addition, as shown in Figure 2, the GEOs can be seen all the day and the ambiguity terms of the GEOs are steadier than that of other satellites, which better fit the observations.

## 5. Discussion and Conclusions

We have presented static and kinematic PPP results in the modes of BeiDou-only, GPS-only and BeiDou/GPS systems, respectively. The visibility of the current BeiDou satellite constellation, multipath combinations and undifferenced phase and code observation residuals of BeiDou satellites are used in the analysis. The results are compared with the “ground truth of stations”. The following conclusions can be drawn:

(1) The analysis of multipath combinations shows that the MPCs of BeiDou code measurements are higher than those of GPS code measurements at the testing stations;
(2) The daily static PPP solutions using BeiDou code and phase measurements demonstrate that the horizontal precision is better than 1.0 cm while the vertical precision is better than 3.0 cm in the Asian-Pacific region. For the region having less observed BeiDou satellites, the horizontal precision of BeiDou-only PPP solutions reaches 3.0 cm. The horizontal precision of BeiDou-only kinematic PPP solutions is better than 3.0 cm and the vertical precision is better than 6.0 cm, which is close to that of GPS-only kinematic PPP. The convergence time of BeiDou-only kinematic PPP is longer than that of GPS-only kinematic PPP;
(3) The daily static PPP solutions using BeiDou/GPS code and phase measurements demonstrate that the horizontal and vertical precisions are better than 1.0 cm, which is close to those of GPS-only static PPP. The horizontal precision of kinematic BeiDou/GPS PPP solutions is better than 2.0 cm while the vertical precision is better than 5.0 cm. The results improve significantly compared to that of BeiDou-only and GPS-only kinematic PPP. The convergence time of BeiDou/GPS kinematic PPP is also shorter than that of BeiDou-only and GPS-only kinematic PPP. The East and North components converge faster than the Up component. And less than 1 hour is used in all the components;
(4) The analysis on the residuals shows that BeiDou undifferenced LC and PC residuals vary with orbital characteristics. The BeiDou code and phase residuals are higher than the GPS ones, which may be due to the multipath error.

Finally, it should be pointed out that the performance evaluation of BeiDou PPP is for preliminary results since the BeiDou constellation is not yet completed. Furthermore, as the number and proper distribution of the ground tracking stations increase, the accuracy of satellite orbit and clock biases will also improve greatly [31]. The precision of BeiDou positioning may be significantly improved in the future. Moreover, the determination and optimization of the phase center corrections in the satellites and receiver terminals may also benefit the performance of BeiDou positioning.

## Figures and Tables

**Table 1.** The strategies for BeiDou PPP and BeiDou/GPS PPP.

**Item**	**Models & Constraints**
Observables	Undifferenced ionosphere-free code and phase combination of B1 and B2
Elevation angle cutoff	7°
Sampling rate	30 s
Precise obit	GPS: IGS Final Ephemeris BeiDou: PANDA BeiDou Final Ephemeris
Precise clock biases	GPS: IGS Precise Satellite Clock biases 30 s BeiDou: PANDA BeiDou Precise Satellite Clock biases 30 s
Satellite Antenna PCO	Default values from manufacturer
Satellite Antenna PCV	Only GPS
Phase rotation correction	Phase polarization effects applied [29]
Receiver Antenna PCO and PCV	Not applied
Troposphere model	Saastamoinen model for wet and dry hydrostatic delay with Global Mapping Function [30]
Ionosphere	1st order effect eliminated by forming ionosphere-free linear combination
Solid Earth tides	IERS Conventions 2010
Ocean tides	IERS Conventions 2010
Solid Earth pole tides	IERS Conventions 2010
Relativistic effects	IERS Conventions 2010
Time system	GPS Time
Terrestrial frame	ITRF2008
Parameters estimation	Model & Constraint
Coordinate	Static mode: constants, 10 m Kinematic mode: estimated as a random walk process for each epoch, 10 m, 10 m/sqrt (h)
Receiver clock biases	Estimated as a random walk process for each epoch, 300 m/sqrt (h)
Troposphere	Initial model and piece-wise constant in 2 h interval, 20 cm, 2 cm/sqrt (h)
Integer ambiguity	Constant for each ambiguity arc
System time difference	Estimated as a random walk process for each epoch, 300 m/sqrt (h)

**Table 2.** Biases of the results of the BeiDou, GPS, and BeiDou/GPS static PPP of the “BETS” against the “ground truth” in the East, North, and Up components on September 24, 2012.

**SITE**	**BeiDou**			**GPS**			**BeiDou/GPS**		

	**East(cm)**	**North(cm)**	**Up(cm)**	**East(cm)**	**North(cm)**	**Up(cm)**	**East(cm)**	**North(cm)**	**Up(cm)**
BJF1	–0.18	–0.05	–1.44	0.66	–0.05	–1.95	–0.23	–0.32	–0.20
CHDU	0.87	0.05	1.50	0.62	–0.31	–0.71	0.69	–0.11	0.62
CWKJ	–0.22	0.51	–3.27	0.43	–0.31	–0.87	–0.24	0.10	–0.88
HRBN	0.86	0.41	–1.37	0.55	–0.36	–-0.79	0.54	–0.10	–0.63
LASA	1.60	0.44	–2.14	1.23	–0.26	–0.16	0.92	0.16	–1.19
WDKJ	0.48	0.28	–2.58	0.31	–0.20	0.01	–0.05	0.27	–0.34
WUHN	–0.6	0.37	–2.68	0.35	–0.51	1.19	0.08	0.08	–1.34
XIAN	0.39	0.40	–2.34	–0.51	–0.40	–1.04	–0.30	0.25	–1.32
LEID	–0.97	–1.78	0.81	0.03	–0.07	0.01	–0.25	0.03	0.31
GREECE	1.04	–0.27	–6.22	0.29	0.07	1.10	0.58	0.14	0.63
RMS	0.79	0.62	2.70	0.56	0.28	0.93	0.45	0.17	0.81

**Table 3.** Average values of the RMS of undifferenced LC and PC residuals for all BeiDou satellites.

**PRN**	**LC(cm)**	**PC(m)**
C01	0.71	1.29
C03	0.67	1.35
C04	0.89	2.02
C05	0.85	1.92
C06	0.84	1.33
C07	1.12	1.37
C08	0.85	1.24
C09	0.86	1.16
C10	0.89	1.25
C11	1.12	1.25
C12	1.62	1.42
Mean	0.95	1.42
